# The Role of Surgical Expertise and Surgical Access in Retroperitoneal Sarcoma Resection – A Retrospective Study

**DOI:** 10.3389/fsurg.2022.883210

**Published:** 2022-05-12

**Authors:** P. Aeschbacher, A. Kollár, D. Candinas, G. Beldi, A. Lachenmayer

**Affiliations:** ^1^ Department of Visceral Surgery and Medicine, Inselspital, Bern University Hospital, University of Bern, Bern, Switzerland; ^2^ Department of Medical Oncology, Inselspital, Bern University Hospital, University of Bern, Bern, Switzerland

**Keywords:** retroperitoneal sarcoma, sarcoma, surgical access, multi-visceral resection, sarcoma resection

## Abstract

**Background:**

Retroperitoneal sarcoma (RPS) is a rare disease often requiring multi-visceral and wide margin resections for which a resection in a sarcoma center is advised. Midline incision seems to be the access of choice. However, up to now there is no evidence for the best surgical access. This study aimed to analyze the oncological outcome according to the surgical expertise and also the incision used for the resection.

**Methods:**

All patients treated for RPS between 2007 and 2018 at the Department of Visceral Surgery and Medicine of the University Hospital Bern and receiving a RPS resection in curative intent were included. Patient- and treatment specific factors as well as local recurrence-free, disease-free and overall survival were analyzed in correlation to the hospital type where the resection occurred.

**Results:**

Thirty-five patients were treated for RPS at our center. The majority received their primary RPS resection at a sarcoma center (SC = 23) the rest of the resection were performed in a non-sarcoma center (non-SC = 12). Median tumor size was 24 cm. Resections were performed via a midline laparotomy (ML = 31) or flank incision (FI = 4). All patients with a primary FI (*n* = 4) were operated in a non-SC (*p* = 0.003). No patient operated at a non-SC received a multivisceral resection (*p* = 0.004). Incomplete resection (R2) was observed more often when resection was done in a non-SC (*p* = 0.013). Resection at a non-SC was significantly associated with worse recurrence-free survival and disease-free survival after R0/1 resection (2 vs 17 months; Log Rank *p*-value = 0.02 respectively 2 vs 15 months; Log Rank *p*-value < 0.001).

**Conclusions:**

Resection at a non-SC is associated with more incomplete resection and worse outcome in RPS surgery. Inadequate access, such as FI, may prevent complete resection and multivisceral resection if indicated and demonstrates the importance of surgical expertise in the outcome of RPS resection.

## Background

Retroperitoneal Sarcoma (RPS) is a rare tumor entity (1 new case/100.000 inhabitants/year) that represents 12%–16% of all soft tissue sarcomas (STS). Its overall 5-year survival varies between 50 and 60% (1–3). Liposarcoma represents 60% of RPS followed by leiomyosarcoma, and less often malignant peripheral nerve sheath tumor, undifferentiated pleomorphic sarcoma, solitary fibrous tumor, and other sarcomas (4, 5). They can be located anywhere in the retroperitoneal space with no clear delimitation making a complete resection a particular challenge (6). Unlike extremity-trunk sarcomas, patients with RPS develop local recurrences (80%) more often than distant metastases even when completely resected (7).

Due to the rarity of this pathology, actual recommendations are often based on a low level of evidence, mainly with expert opinions and retrospective studies with low patient numbers (3, 5, 8–11). Chemotherapy and radiation therapy, despite controversial opinions, are playing a growing role in the neoadjuvant, adjuvant, and palliative treatment of RPS (1, 3, 4, 12). However, a recent multicenter randomized analysis showed no benefit in preoperative radiotherapy (13). Aggressive gross surgical resection remains the treatment of choice and the only curative therapy available (1, 12).

Preoperative assessment is usually done through contrast tomography (CT) and/or magnetic resonance imaging (MRI) (5, 10). An image-guided percutaneous core biopsy might be necessary if the imaging is not typical or neoadjuvant treatment is envisaged (5, 14). A laparoscopic or open biopsy is not recommended because of the risk of abdominal spreading (3, 5). In any way, each case should be discussed in a multidisciplinary team meeting (MDT) of a specialized reference center to determine diagnostic tools and treatments (3, 8, 9).

Current literature recommends to perform a radical and wide excision to ensure a complete en-bloc resection of the tumor. Due to the often unclear borders to the surrounding healthy tissue and adjacent organs, multi-visceral resections (e.g. nephrectomy, colectomy, etc.) are often required to ensure such a complete resection (3, 5). Marginal, non-visceral resections may however remain appropriate in well differentiated pathology. Although these recommendations do not specifically state what incision to use, the midline incision seems to be the access of choice if wide margins are anticipated (14, 15). A thoracic or lumbar extension may be necessary to assure a better exposure or better vascular control of the vena cava inferior and left atrium (14, 16, 17). The midline laparotomy (ML) allows exposure of the intraperitoneal and retroperitoneal compartment while flank incisions (FI) are limited only to the retroperitoneal space not allowing full exposure of the intraperitoneal compartment often necessary for a multi-visceral resection (18).

Even though different surgical accesses might influence the surgical and oncological outcomes of the patient, almost no study reports the access used for RPS resection. One paper recommends performing a generous laparotomy without specifically discussing a limited retroperitoneal access and another one reports midline incision as the most common access (14, 15). In our experience small RPS are sometimes resected through a FI or laparoscopically by surgeons not specifically trained for sarcoma surgery. The FI is typically used for complete or partial nephrectomy or for vascular surgery with the advantage of not opening the peritoneum but does not allow the resection of intraperitoneal structures if necessary (14). In addition, it has been reported that minimal invasive approaches for RPS resections are not recommended (19).

Having observed several cases of early tumor recurrence or remaining tumor after RPS resections through a FI in patients operated at a non-SC, we aimed to analyze the role of the incision with the oncological outcome by analyzing our own sarcoma database.

## Methods

All patients with RPS treated at the Department of Visceral Surgery and Medicine of the University Hospital of Bern, Switzerland, between 2007 and 2018 and receiving a primary RPS resection in curative intent were retrospectively included in the analysis. Data sampling and analysis was performed in August 2020. The study protocol was approved by the Regional Ethical Review Board (KEK-No. 2019-00324).

### Patient Population

At the Department of Visceral Surgery and Medicine, patients were always discussed in the MDT before treatment. Depending on the anatomic location of the tumor and its proximity to neighboring organs, a primary surgical resection was decided. Written informed consent was obtained before the procedure in patients aged 18 or older.

### Data Analysis

Clinical data were extracted from the patient’s records available in the department’s medical system. All patients with surgical resection of their RPS were included in the analysis. We excluded patients who presented with other non retroperitoneal sarcomas. Patients with metastasic disease at the time of the diagnosis, unresectable tumor or ineligible for an operation were excluded. The following data were collected: age, sex, hospital type (sarcoma vs non-sarcoma center), presence of a primary multi-visceral resection, sarcoma subtype, tumor grading, neo/-adjuvant chemotherapy or radiotherapy, postoperative complications, postoperative mortality, presence of local recurrence or metastasis, and recurrence-free (after R0/1 resection), disease-free (after R0/1 resection) and overall survival.

The histological subtypes of RPS were dedifferentiated liposarcoma, well-differentiated liposarcoma or leiomyosarcoma. The tumors histology was graded from 1 (low-grade) to 3 (high-grade) according to the FNCLCC (Fédération Nationale des Centres de Lutte Contre Le Cancer). This classification takes in account cells differentiation, mitotic count score and tumor necrosis score (9).

The primary resection was considered as complete (R0/1) when there was no microscopical evidence of tumor at the margin or when the resection was macroscopically complete with microscopically positive margins (R1). Incomplete resections included macroscopically and microscopically incomplete (R2) resections. Studies on the subject also usually make a grouping of R0/1 vs R2. Tumor tissue found on imaging after R2 resection was considered as residual.

Postoperative complications after the surgical procedure were classified according to the Clavien-Dindo classification (20). Clavien-Dindo grade ≥IIIb was considered as a major postoperative complication as it required a reintervention under general anesthesia.

Recurrence-free survival is defined as the time in months from the first RPS resection to the first local recurrence for patients with a R0/1 resection. Progression-free survival is defined as the time in months from the first RPS resection until progression is detected after incomplete resection (R2). Disease-free survival is defined as the time in months from the primary RPS resection to the first local recurrence, progression and/or metastasis. Overall survival is defined as the time in months from the first RPS resection to the time of death or last follow-up.

### Statistics

We applied descriptive statistics for the presentation of clinical and outcome data. Continuous data are shown as median and interquartile range (IQR) where appropriate. The Chi-square or Fisher's exact test, and the Mann-Whitney U test were used to compare categorical and continuous variables, as appropriate. The Kaplan-Meier method, log rank test and logistic regression were applied to analyze the association of variables with local recurrence, progression-free, and overall survival. Cox regression for recurrence/local disease progression was performed with the following variable: access type, resection type, tumor size (median of 24 cm was used as the cutoff), resection margin, histopathology and FNCLCC grading. The threshold for statistical significance was set to *p*-value ≤ 0.05. Descriptive statistics and graphs were analyzed using SPSS Version 25.

## Results

### Clinical Data

From January 2007 to December 2018, 35 patients were treated for a RPS at our center and received a primary RPS resection in curative intent. Patients were either operated on at our SC (*n* = 22) or another SC (*n* = 1) or were operated on in a hospital not recognized as a SC and then transferred for further management to our center (*n* = 12). Gender, median age and tumor size were not significantly different between the two groups (Table 1).

**Table 1:** Clinical data of patients undergoing surgery for retroperitoneal sarcoma according to the type of hospital where the resection was performed.

Variable	Resection at a sarcoma center *n* (%) or median (IQ-range) *N* = 23	Resection at a non-sarcoma center *n* (%) or median (IQ-range) *N* = 12	*p*-value^a^
Age	61 (47;73)	68 (62;73)	0.091
Men	10 (44)	9 (75)	0.076
Midline laparotomy	23 (100)	8 (67)	**0**.**003**
Flank incision	0 (0)	4 (33)	
Primary multi-visceral resection	11 (48)	0 (0)	**0**.**004**
Tumor size (cm)	25 (17;30)	17 (8;24)	0.091

^*a*^ *Fisher's exact test and the Mann-Whitney U test were used to compare categorical and continuous variables, as appropriate.*

All patients with a primary FI (*n* = 4) were operated in a non-SC (*p* = 0.003). No patient operated at a non-SC received a multivisceral resection (*p* = 0.004) (Table 1). Only one patient in the FI group was discussed in the MDT prior to the initial resection and a treatment at a SC was recommended but not performed. Two patients were resected through a FI although the diagnosis of sarcoma was already known through a preoperative biopsy. A nephrectomy was performed in each of these two patients with a concurrent retroperitoneal lymphadenectomy in one patient.

### Histopathological Data and Resection Status

Details on the histological type, FNCLCC grade and perioperative chemo- and radiotherapy are reported in Table 2. Macroscopically complete resections (R0/1) were achieved in 27 (77%) patients, 21 (78%) at a SC and 6 (22%) at a non-SC. Seven patients (20%) had an incomplete resection (R2), 2 (29%) at a SC and 5 (71%) at a non-SC. For one patient, this information was not available, neither in the histopathological or operation report. Incomplete resection (R2) was observed more often when resection was done in a non-SC (*p* = 0.013) (Table 2).

**Table 2:** Perioperative data of patients undergoing surgery for retroperitoneal sarcoma according to the type of hospital where the resection was performed.

Variable	Resection at a sarcoma center *n* (%) *N* = 23	Resection at a non-sarcoma center *n* (%) *N* = 12	*p*-value^a^
**Histopathology**			
Dedifferentiated liposarcoma (DDLS)	10 (44)	6 (50)	0.818
Well-differentiated liposarcoma (WDLS)	7 (30)	4 (33)	
Leiomyosarcoma	6 (26)	2 (17)	
**FNCLCC Grading**			
Grade 1	7 (30)	3 (25)	0.520
Grade 2	8 (35)	7 (58)	
Grade 3	6 (26)	2 (17)	
Unknown	2 (9)	0 (0)	
**Resection margin**			
R0/1	21 (91)	6 (50)	**0.013**
R2	2 (9)	5 (42)	
Unknown	0 (0)	1 (8)	
Postoperative Chemotherapy	0 (0)	0 (0)	
Postoperative Radiotherapy	4 (17)	0 (0)	0.125
Intraoperative radiotherapy	4 (17)	0 (0)	0.125
Clavien Dindo Classification Grad < 3b	3 (13)	3 (25)	0.373
Clavien Dindo Classification Grad ≥ 3b	3 (13)	0 (0)	0.191
Postoperativ mortality (within 30 days)	1 (4)	0 (0)	0.464
Local recurrence (after R0/1)	12 (57)	5 (83)	0.241
Local disease progression (after R2)	0 (0)	4 (80)	0.053
Overall early local disease progression (<3 months)	0 (0)	2 (17)	**0.044**
Metastasis	6 (26)	2 (17)	0.529

*^a^* *Fisher's exact test.*

### Morbidity and Mortality

The overall postoperative morbidity was 26% (9 patients) with 9% (3 patients) having a major postoperative complication (Clavien Dindo ≥ IIIb) (Table 2). Complications consisted of (1) Vena cava thrombosis after vena cava replacement with xenopericardium patch with the need for operative revision and thrombectomy, (2) Intestinal anastomosis insufficiency leading to pulmonary embolism, and pneumonia with septic shock and (3) Multiorgan failure due to a pancreatic fistula, with intraabdominal abscess leading to death. Although severe postoperative morbidity was higher at a SC, this reached no statistical significance (13% vs. 0% *p* = 0.191).

### Local Recurrence and Metastases

Local recurrence after R0/1 resection occurred in 57% (12/21) after resection at a SC and in 83% (5/6) after resection at a non-SC (*p* = 0.241). Local disease progression after R2 resection was detected in 4 patients after resection at a non-SC. No disease progression was observed after R2 resection at a SC (*p* = 0.053). Early (<3 months postoperatively) recurrence or disease progression occurred in 2 patients after resection at a non-SC (*p* = 0.044) (Table 2).

**Figure 1:**
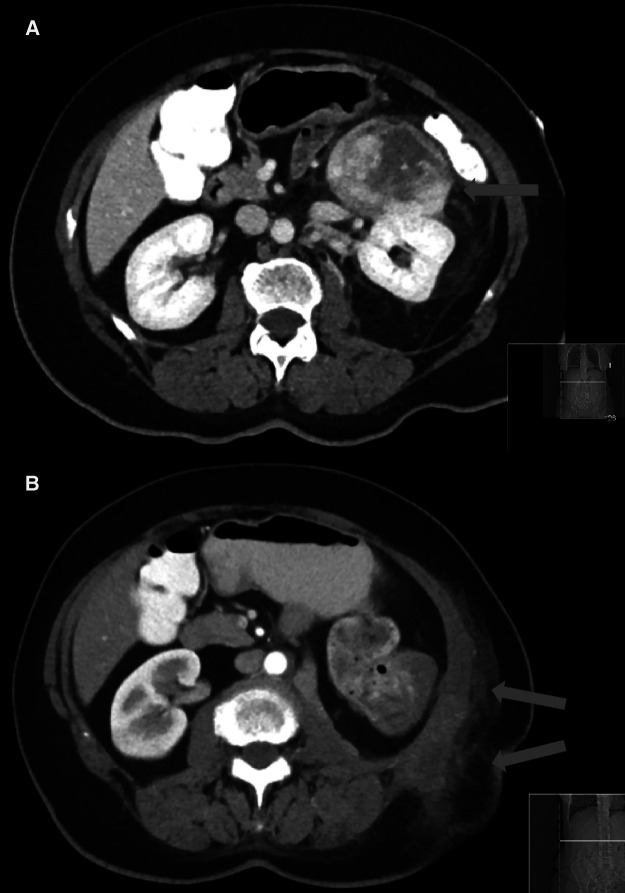
Computer tomography of a retroperitoneal sarcoma (**A**) prior the resection through a flank incision and (**B**) the local recurrence at one month after the resection confirmed with a biopsy.

One of the four patients operated through a FI had a R2 resection with postoperative radiotherapy for the remaining tumor in the flank, the abdominal fascia and in the incision line (Figure 1) requiring a second resection at our institution with a costal, diaphragm, and musculus quadratus lumborum resection. The patient stayed recurrence-free for two years afterwards. Another patient operated through the FI resulting in a R2 situation should have had a multi-visceral resection which could not be performed through the limited access despite the visible tumor on the mesocolon. For the third patient of the FI group, the resection also resulted in a documented R2 situation and a progression visible on imaging occurred 16 months afterwards. A percutaneous radiotherapy was then performed followed by a resection attempt showing a tumor that was not surgically removable anymore. The fourth patient of that group was R1 resected and recurred locally after two months requiring a multi-visceral resection with intraoperative radiotherapy.

Eight patients (26%) developed distant metastases, of whom four patients also had a local recurrence. There was no difference in the rate of metastasis occurrence when the patient was operated at a SC or non-SC (*p* = 0.529) (Table 2).

### Survival Analysis

**Figure 2:**
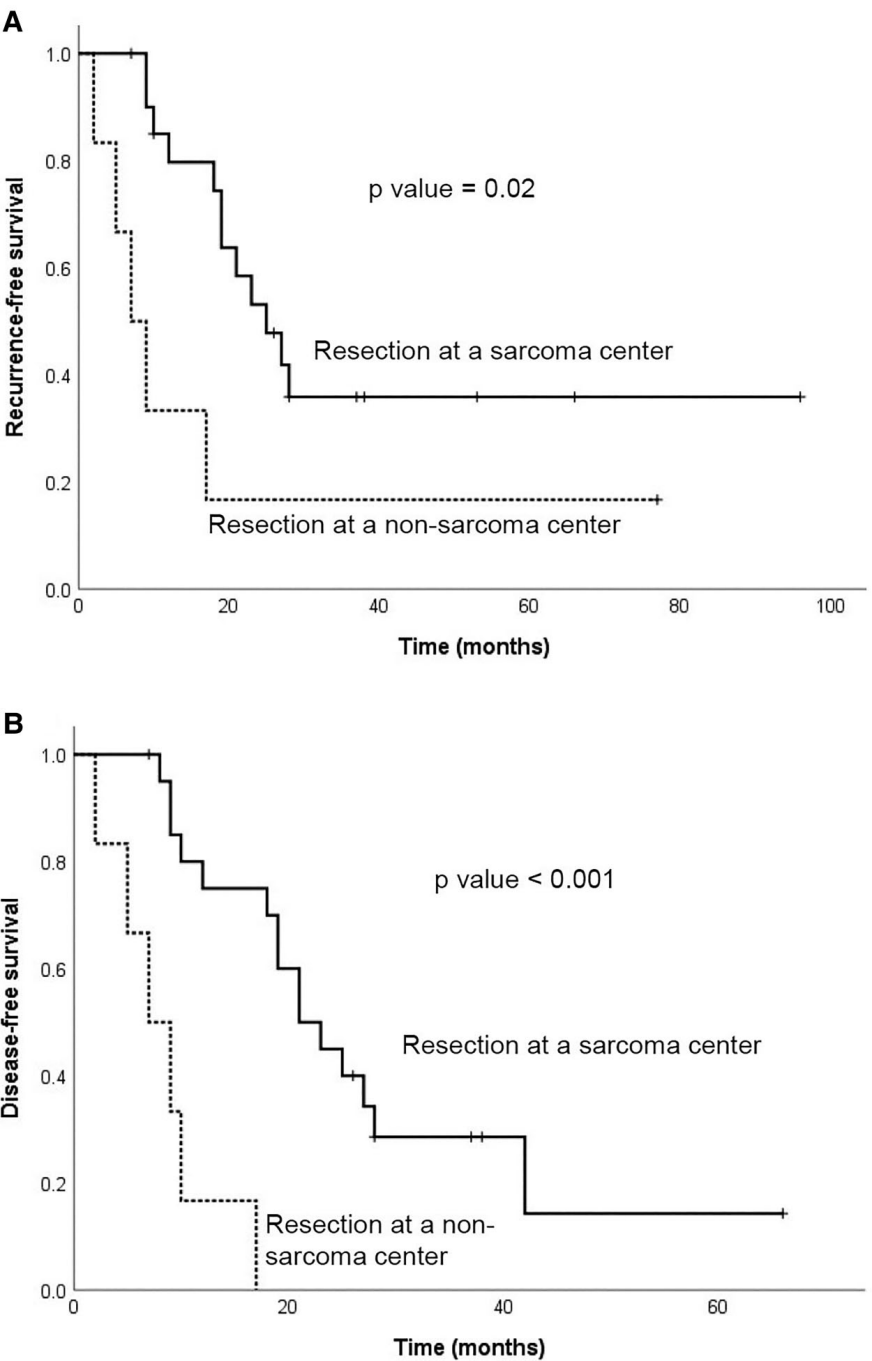
Kaplan-Meier curve for (**A**) recurrence-free survival and (**B**) disease-free survival after R0/1 resection according to the type of hospital where the resection was performed (sarcoma center vs non-sarcoma center).

Median recurrence-free survival after R0/1 resection was 21 months and 3-years recurrence-free survival was of 32%. Median disease-free survival after R0/1 resection was 19 months and 3-years disease-free survival 22%. Resection at a non-SC was significantly associated with worse recurrence-free survival and disease-free survival after R0/1 resection (2 vs 17 months; log rank *p*-value = 0.02 respectively 2 vs 15 months; log rank *p*-value < 0.001) (Figures 2A, B**)**. A progression/recurrence-free survival Cox regression analysis showed that resection through a ML (*p*-value = 0.017, HR = 0.024) was associated with longer progression/recurrence-free survival while bigger tumor size (*p*-value = 0.019, HR 6.911) was associated with shorter progression/recurrence-free survival (Table 3, Supplementary Figures 1a,b).

**Table 3:** Cox Regression analysis for recurrence/local disease progression.

Variable	*p*-value	HR	95% CI
Midline laparotomy vs Flank incision	**0** **.** **017**	**0** **.** **024**	**0.001–0.508**
Primary multi-visceral resection	0.317	2.098	0.492–8.955
Tumor size (≤24 cm vs >24 cm)	**0** **.** **019**	**6** **.** **911**	**1.372–34.807**
Resection margin	0.279	5.974	0.235–151.656
Histopathology	0.627	1.320	0.431–4.044
FNCLCC Grading	0.130	2.412	0.771–7.543

The median overall survival was 77 months at a median follow-up time of 40 months and 3-years overall survival was 86%. There was no significant difference between survival after resection at a SC or non-SC (*p* = 0.893). Two patients were lost to follow-up.

## Discussion

Our retrospective study compares a population of patients with RPS sarcoma undergoing a resection at our SC or at a non-SC. Multi-visceral resection was never performed at a non-SC (*p* = 0.004) and we observed there RPS resection through a flank incision (*p* = 0.003). Resection at non-SC also had more R2 resection (*p* = 0.013) and early local disease progression (*p* = 0.044). Flank incision at non-SC may be a reason for poorer surgical and oncological outcome especially due to incomplete resection (R2) and the impossibility of multivisceral resection.

This retrospective study of 35 patients is clearly limited due to its small sample size but represents typical RPS patients with a median age of 62 years and median tumor size of 24 cm at the time of diagnosis (1, 21, 22). Pathology reports also revealed a typical mixture of RPS with the majority being liposarcoma followed by leiomyosarcoma (1, 21).

We report a R0/1 rate of 91% and a R2 rate of 9% for RPS resection at a SC, which reflects the rates reported in the current literature (73%–94% and 9%–29%, respectively) (1, 22, 23). In contrast, patients resected at a non-SC had a R2 rate of 42% resulting in a significantly higher early recurrence or local disease progression rate, ultimately leading to worse survival (1).

Our article mentions the type of access as an important element in the resection of a RPS. The importance of a complete surgical excision including multi-visceral resections whenever necessary has already been proven for RPS patients (10, 12, 24). Gronchi et al. and Bonvalot et al. presented two retrospective series showing that an initial aggressive surgical approach provides a better oncological outcome (1, 22). Based on these results wide margin excisions with en-bloc resection are recommended in guidelines of the Trans-Atlantic RPS Working Group and French ccAFU guidelines for the treatment of RPS (3, 9, 15). Although a ML with a possible thoracolumbar extension in case of complex vascular procedures seems to be the access of choice to perform wide resections, many patients are still being resected through a restricted retroperitoneal access not allowing multi-visceral resections. Neither guidelines nor the literature give a clear recommendation to what surgical access should be used. Another problematic in the resection of RPS is the use of laparoscopic surgery.

One recent retrospective study reported similar overall survival and postoperative mortality in minimal invasive RPS resection compared to open resection. However, minimal invasive resection was mainly performed for tumors of smaller size and the study reported no data on recurrence-free survival. Minimal invasive resection could be a limiting factor for a radical resection of the RPS, similar to a flank access. Due to the lack of evidence and the complexity of such resection with an often unclear tumor delimitation, a minimal invasive RPS resection is not recommended in this rare disease (19, 25).

As known from the literature, tumor size was also associated with shorter recurrence-/disease progression-free survival in our analysis. Interestingly, the tumor size did not differ between the access type with even an early recurrence of the smallest tumor of 4 cm two months after a resection through a FI.

Recent studies reported that RPS resections in SCs after discussion in a multidisciplinary team meeting show better oncological and surgical outcomes (10, 26–28). In line with these results, our recurrence-free survival was longer for patients treated at a SC compared to those operated in non-SC after excluding incomplete resection (R2). In addition, we observed that patients initially treated in a non-SC almost never received multi-visceral resections suggesting a limited local experience with this rare tumor entity. It has been shown that RPS resections performed in SCs with high expertise in visceral RPS surgery and peri-operative management of multi-visceral resections have better outcomes (26, 29, 30). Compliance to current practical guidelines is reported to be significantly better in specialized reference centers supported by data showing that patients are often not discussed in a mandatory multidisciplinary team meeting in the real-life practice (3, 31).

Although high-grade postoperative complications were slightly higher for resection at a SC, our postoperative morbidity and mortality rates remain low in comparison to the literature (32). More aggressive surgical approaches for RPS resection are known to provide an oncological benefit without increasing mortality and morbidity; or a worsening of the oncological outcome after surgical complications (1, 22, 24).

In our cohort, we could show that FI and bigger tumor size were associated with shorter recurrence/disease progression-free survival in the Cox regression analysis. However, the conclusions should be taken with caution, as the sample size is very small. We were not able to confirm other factors known to be associated with poor recurrence-free survival such as age, multifocality, the extent of resection, number of organs resected, radiotherapy and chemotherapy (23, 26). In comparison to the literature where the recurrence-free survival is estimated at 41%–59%, our study presents a slightly lower recurrence-free survival of 32% at 3 years. Our 3-years disease-free survival of 22% is also shorter compared with the 34%–79% at 5-years reported in the literature (1, 2, 21, 33).

The role of radiotherapy on disease-free survival and recurrence-free survival is still unclear for the treatment of RPS (34), but the postoperative radiotherapy performed (12.9%) could have influenced the oncological outcome after resection at a SC.

Due to the small sample size of our study, we were not able to detect any overall survival differences between the two access groups and the type of hospital. Our 5-years overall survival of 62% is similar to what literature reports (36%–60%) (1, 21–23, 32).

It is possible that the population referred to us from a peripheral hospital for management following resection presents a selection bias in that this population is mostly represented by complex cases with insufficient resection. However, every patient with the diagnosis of sarcoma should be referred to a SC for evaluation at an MDT and follow-up. Therefore, we think that this bias remains low.

Clearly, the small sample size is a limitation of our study which still has a descriptive value in that it highlights a problem encountered in the management of patients with RPS and in the context of a lack of studies on this subject. Results should be taken with caution as the sample is small and that most tests do not have a strength greater than 80%. However, this is justified with the relatively low incidence of these tumors even though a 12-year range was considered. This problem may seem trivial for a surgeon with expertise in the treatment of sarcoma where an approach allowing multivisceral resection seems obvious. However, it is clear that this element is almost never mentioned in the recommendations and studies and is not systematically applied. RPS resection through a flank access is probabely closely linked with a lack of surgical expertise and a lack of radicality of the resection and it is possible that these last two elements play a predominant role in the outcome of these resections.

## Conclusions

Resection at a non-SC is associated with more incomplete resection and worse outcome in RPS surgery. Inadequate access, such as FI, may prevent complete resection and multivisceral resection if indicated and demonstrates the importance of surgical expertise in the outcome of RPS resection. Treatment at non-SC should be avoided in order to improve the outcome of patients. However, stronger studies on the subject are necessary.

## Data Availability

The raw data supporting the conclusions of this article will be made available by the authors, without undue reservation.
