# Supplementary figures and images for: The Role of Surgical Expertise and Surgical Access in Retroperitoneal Sarcoma Resection – A Retrospective Study
Source: Front Surg. 2022 May 12;9:883210. doi: 10.3389/fsurg.2022.883210 (PMC9133808; doi:10.3389/fsurg.2022.883210)

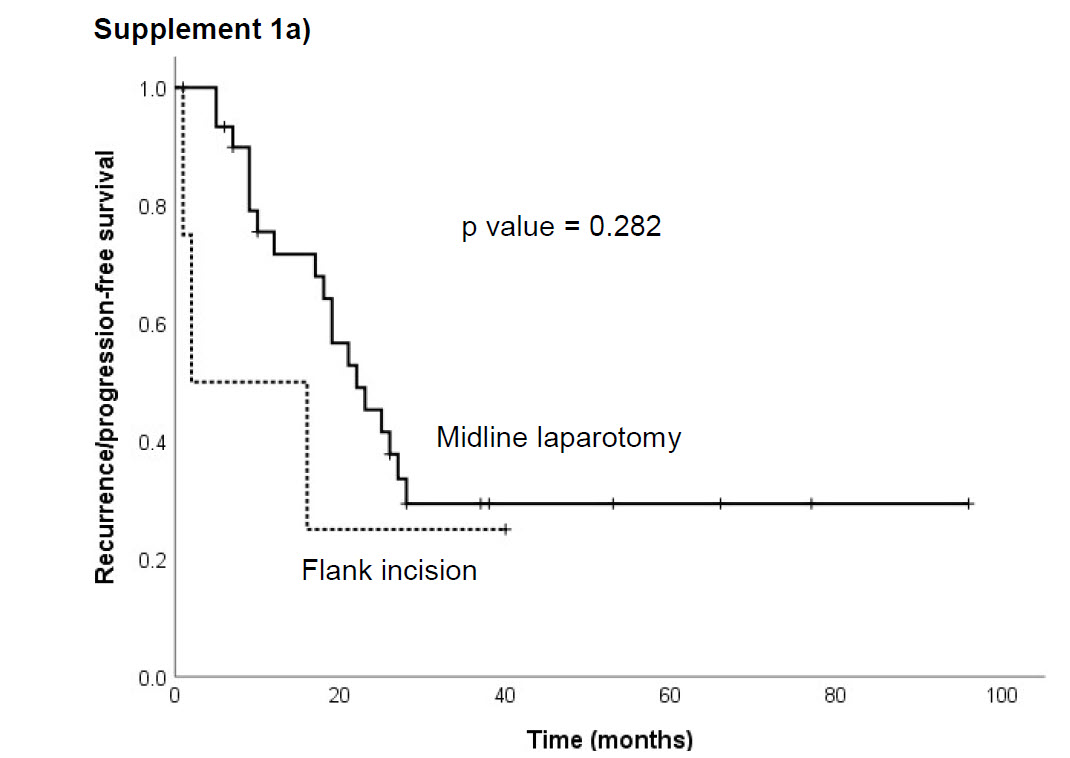

Supplement: Supplementary file 1 [file Image_1_v1.jpeg]

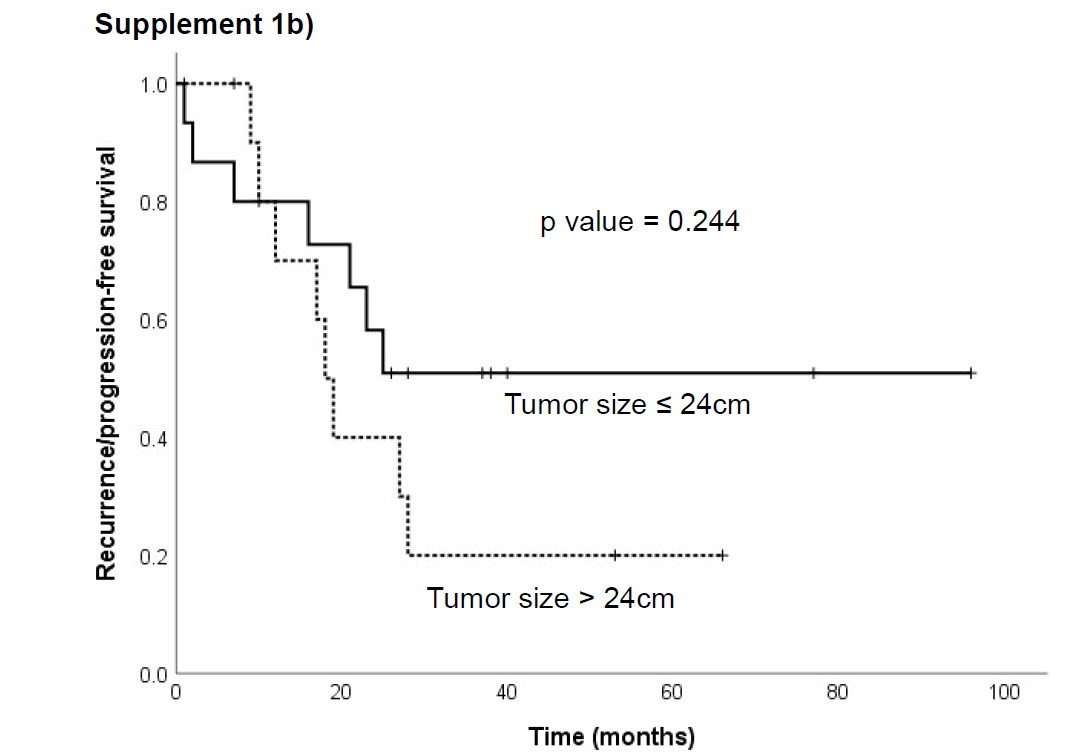

Supplement: Supplementary file 2 [file Image_2_v1.jpeg]
